# mTOR Signaling in Pulmonary Vascular Disease: Pathogenic Role and Therapeutic Target

**DOI:** 10.3390/ijms22042144

**Published:** 2021-02-21

**Authors:** Aleksandra Babicheva, Ayako Makino, Jason X.-J. Yuan

**Affiliations:** 1 Section of Physiology, Division of Pulmonary, Critical Care and Sleep Medicine, Department of Medicine, University of California, San Diego, CA 92093, USA; ababicheva@health.ucsd.edu; 2 Division of Endocrinology and Metabolism, Department of Medicine, University of California, San Diego, 9500 Gilman Drive, MC 0856, La Jolla, CA 92093-0856, USA; amakino@health.ucsd.edu

**Keywords:** RTK/PI3K/AKT/mTOR pathway, Raptor, Rictor, SMC transition, EndMT

## Abstract

Pulmonary arterial hypertension (PAH) is a progressive and fatal disease without a cure. The exact pathogenic mechanisms of PAH are complex and poorly understood, yet a number of abnormally expressed genes and regulatory pathways contribute to sustained vasoconstriction and vascular remodeling of the distal pulmonary arteries. Mammalian target of rapamycin (mTOR) is one of the major signaling pathways implicated in regulating cell proliferation, migration, differentiation, and protein synthesis. Here we will describe the canonical mTOR pathway, structural and functional differences between mTOR complexes 1 and 2, as well as the crosstalk with other important signaling cascades in the development of PAH. The pathogenic role of mTOR in pulmonary vascular remodeling and sustained vasoconstriction due to its contribution to proliferation, migration, phenotypic transition, and gene regulation in pulmonary artery smooth muscle and endothelial cells will be discussed. Despite the progress in our elucidation of the etiology and pathogenesis of PAH over the two last decades, there is a lack of effective therapeutic agents to treat PAH patients representing a significant unmet clinical need. In this review, we will explore the possibility and therapeutic potential to use inhibitors of mTOR signaling cascade to treat PAH.

## 1. Introduction

Idiopathic pulmonary arterial hypertension (IPAH) is a progressive and fatal disease in which functional and structural changes in the pulmonary vasculature lead to the increase in pulmonary vascular resistance (PVR) and pulmonary arterial pressure (PAP) [1,2]. Patients with IPAH, if untreated, die mainly because of progressive right heart failure, and the response of the right ventricle (RV) to the increased afterload is an important determinant of outcome in patients [3,4,5].

Regardless of the initial pathogenic trigger, sustained pulmonary vasoconstriction, concentric pulmonary vascular remodeling, in situ thrombosis, and increased pulmonary artery (PA) wall stiffness all directly contribute to increasing PVR and PAP in patients with pulmonary arterial hypertension (PAH) and animal with experimental pulmonary hypertension (PH) [6,7]. Concentric PA wall thickening is characterized by significant intimal, medial, and adventitial thickening due to: (i) increased proliferation of pulmonary vascular endothelial cells (EC), PA smooth muscle cells (PASMC), and fibroblasts (FB), (ii) inhibited apoptosis of EC and PASMC, and (iii) increased endothelial-to-mesenchymal transition (EndMT) that converts EC to proliferative myofibroblast (myoFB), a mesenchymal cell with functional and structural characteristics in common with FB and synthetic or proliferative SMC. Enhanced PASMC proliferation contributes to the concentric PA medial thickening. The phenotypical transition of slowly growing EC to proliferative myoFB, via enhanced EndMT, contributes to neointimal formation, intimal thickening, and distal PA obliteration [8,9,10]. The concentric PA wall thickening and intraluminal obliteration in small arteries and arterioles directly increase PVR and RV afterload in PAH patients.

Multiple cellular and molecular mechanisms are implicated in the development and progression of pulmonary vascular remodeling through enhanced PASMC proliferation and migration, such as the signaling cascades involving increases in intracellular free [Ca^2+^], downregulation of K^+^ channels and bone morphogenetic protein (BMP) receptors, upregulation of transforming growth factor-β (TGF-β) receptors, membrane receptors (e.g., Notch and CaSR) and Ca^2+^-permeable cation channels, and phosphorylation or activation of cytoplasmic kinases like AKT and mammalian target of rapamycin (mTOR) [11,12,13,14,15,16,17,18,19,20]. However, the specific sequence of events involved in the enhanced PASMC proliferation in PH remains unclear.

When cells, including PASMC, are activated by extracellular mitogenic factors such as platelet-derived growth factor (PDGF), epidermal growth factor (EGF), fibroblast growth factor (FGF), insulin-like growth factor (IGF), and endothelin-1 (ET), the AKT/mTOR signaling pathway plays an important role in regulating cell proliferation, migration, and apoptosis, much of which has been linked to subsequent activation of the mTOR, an important downstream signaling protein. In this review, we will focus on the pathogenic role of the mTOR signaling in the development and progression of PAH and also discuss the possibility and potential to use specific inhibitors of mTOR signaling cascade to treat PAH.

### 1.1. The Canonical PI3K/AKT/mTOR Signaling

**Figure 1:**
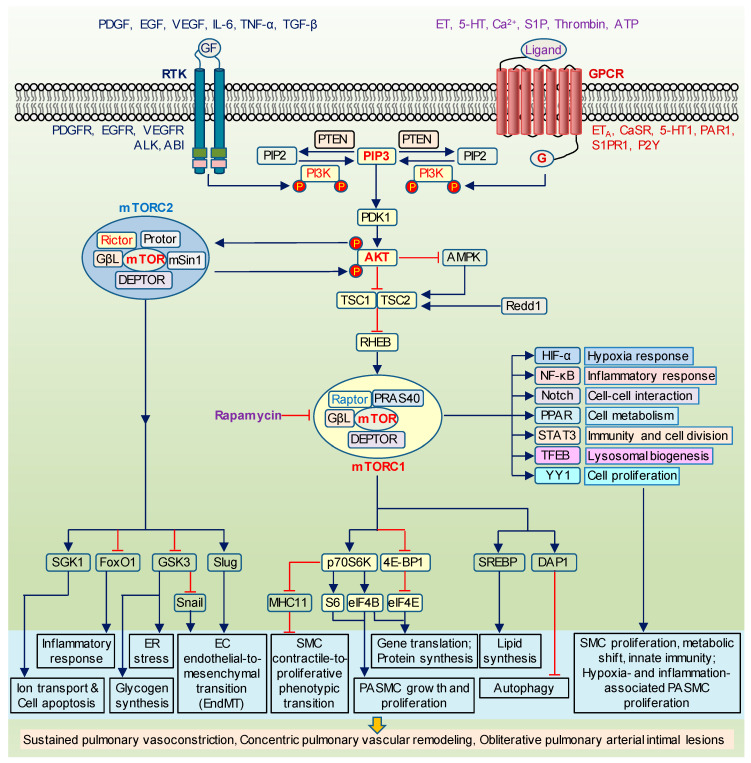
The canonical PI3K/AKT/mTOR signaling. Schematic diagram depicting the canonical PI3K/AKT/mTOR signaling cascade and its proposed effect on cell proliferation, inflammation, metabolism, and gene expression. Upon the activation of receptor tyrosine kinases (RTK) and/or G protein-coupled receptors (GPCR) by mitogenic ligands and growth factors (GF), p85 subunit of PI3K binds to RTK while GPCR activate Ras. Both mechanisms stimulate PI3K/AKT/mTOR signaling. AMPK and Redd1 inhibit mTORC1 via the promotion of TSC1/TSC2 assembly. TSC1/TSC2 complex, in turn, suppresses a direct stimulator of mTORC1, Rheb. The upregulated mTORC1 regulates a number of downstream targets including p70S6K (ribosomal S6 kinase), 4E-BP1 (4E binding protein 1), SREBP (sterol regulatory element binding protein), DAP1 (death-associated protein 1) to enhance protein synthesis, cell proliferation, autophagy, and PASMC phenotypical switch from contractile to proliferative phenotype. It also has a crosstalk with other major signaling such as HIF (hypoxia-inducible factor), NF-κB (nuclear factor kappa B), Notch, PPAR (peroxisome proliferator-activated receptor), STAT3 (signal transducer and activator of transcription 3), TFEB (bHLH leucine zipper transcription factor EB), and YY1 (yin-yang 1). The downstream targets of mTORC2 include GSK3 (glycogen synthase kinase 3), SGK1 (serum- and glucocorticoid-induced protein kinase 1), FoxO1 (Forkhead box O1), Snail, Slug to control glycogen production, ion channels activity, apoptosis, inflammation, and endothelial transition to mesenchymal phenotype. Rapamycin is able to inhibit mTORC1. PI3K, phosphoinositide 3-kinases; PIP2, phospholipid PI(4,5)P2; PIP3, phospholipid PI(3,4,5)P3; PTEN, phosphatase and tensin homologue; PDK1, serine/threonine kinase-3′-phosphoinositide-dependent kinase 1; TSC1/2, tuberous sclerosis complex 1/2; AMPK, adenosine monophosphate-activated protein kinase; Redd1, DNA damage response 1; Rheb, GTP binding protein Ras homolog enriched in brain; MHC11, myosin heavy chain 11; PDGF, platelet-derived growth factor; EGF, epidermal growth factor; VEGF, vascular endothelial growth factor; IL-6, interleukin 6; TNF-α, tumor necrosis factor alpha; TGF-β, transforming growth factor beta; PDGFR, PDGF receptors; EGFR, EGF receptors; VEGFR, VEGF receptors; ALK, anaplastic lymphoma kinase; ABl, tyrosine-protein kinase ABl; ET, endothelin; 5-HT, serotonin; S1P, sphingosine-1-phosphate; ATP, adenosine triphosphate; ET_A_, ET receptor A; CaSR, calcium-sensing receptor; 5HT1, 5HT receptor 1; PAR1, protease-activated receptor 1; S1PR1, S1P receptor 1; P2Y, purinergic G protein-coupled receptors.

The PI3K/AKT/mTOR signaling pathway is an important intracellular transduction system involved in regulating cell proliferation, cell differentiation, gene expression, and protein synthesis. The family of lipid kinases termed phosphoinositide 3-kinases (PI3K, also known as phosphatidylinositol 3-kinases) is able to phosphorylate the 3′-hydroxyl group of the inositol ring of phosphatidylinositol. PI3Ks are divided into three different classes (I-III) based on their structure, function, and substrate specificities and could be activated by G protein-coupled receptors (GPCR) and receptor tyrosine kinases (RTK) [21,22]. Upon binding of the p85 regulatory subunit of PI3K to RTK or GPCR on the cell membrane, the p110 catalytic subunit is released, translocated to the plasma membrane, and catalyzes the conversion (phosphorylation) of the phospholipid PI(3,4,5)P3 (PIP3) from the substrate the phospholipid PI(4,5)P2 (PIP2). This activity of PI3K is negatively regulated by phosphatase and tensin homologue (PTEN), which dephosphorylates PIP3 back to PIP2 and prevents further signal transduction (Figure 1). Loss of PTEN is one of the major mechanism for the excessive activation of PI3K signaling leading to the development of cancer [23,24]. The second messenger PIP3 then recruits protein serine/threonine kinase-3′-phosphoinositide-dependent kinase 1 (PDK1) and AKT to the membrane. AKT (also known as protein kinase B) is a serine/threonine kinase with three isoforms (AKT 1-3). After the recruitment, AKT is undergoing conformational changes exposing two critical amino acids residues for phosphorylation. PDK1 phosphorylates AKT at T308 site located in central catalytic domain whereas PDK2 phosphorylates AKT at S473 site located at C-terminal domain. Both phosphorylation events are necessary for the complete AKT activation.

Recently identified potential PDK2 kinases, i.e., DNA-dependent protein kinase (DNA-PK) and mTOR, are considered as Class IV PI3Ks. mTOR serves as a core component of two multi-protein distinct complexes: mTOR complex 1 (mTORC1) that is highly sensitive to rapamycin, insulin, and growth factors, and mTOR complex 2 (mTORC2) that is mostly insensitive to rapamycin. In both complexes, mTOR acts as serine/threonine protein kinase involved in the regulation of cell proliferation, survival, transcription, and protein synthesis, while in mTORC2, it also functions as tyrosine protein kinase, which promotes activation of insulin receptors and controls the actin cytoskeleton. mTOR complexes (mTORC1 and mTORC2) consist of the shared and unique components. The shared components include catalytic subunit mTOR, mammalian lethal with SEC-13 protein 8 (mLST8, also known as G-protein β subunit-like or GβL), and DEP domain containing mTOR-interacting protein (DEPTOR). The regulatory-associated protein of mammalian target of rapamycin (Raptor) and proline-rich AKT substrate 40kDa (PRAS40) are established as the unique proteins for mTORC1, while rapamycin-insensitive companion of mTOR (Rictor), mammalian stress-activated map kinase-interacting protein 1 (mSin1) and protein observed with Rictor 1 and 2 (Protor1/2) are found in mTORC2 complex. Unlike mTORC1, mTORC2 is protected from the inhibitory effect of rapamycin indicating the difference between Raptor-bound mTOR and Rictor-bound mTOR. Nevertheless, the long-term treatment of rapamycin in some cell types appears to inhibit free mTOR before it is assembled into mTORC2 complex [25,26].

The interaction between mTOR and its upstream effector AKT are complex and, somewhat, paradoxical. mTORC1, which is better characterized than mTORC2, is a well-known target of AKT. AKT suppresses a key indirect regulator of mTORC1, the tuberous sclerosis complex 1/2 (TSC1/TSC2, also known as hamartin). The TSC1/TSC2 heterodimer, in turn, blocks mTORC1 by inactivating the GTP binding protein Ras homolog enriched in brain (Rheb), which directly phosphorylates mTORC1 [27,28] (Figure 1). Additionally, the stress and hypoxia response genes adenosine monophosphate-activated protein kinase (AMPK) and Redd1 (DNA damage response 1, also known as RTP801/Dig2/DDIT4) can promote assembly of TSC1/TSC2 resulting in suppressive effect on mTORC1 pathway. Moreover, AMPK is able to phosphorylate Raptor at two inhibitory sites [29]. However, AMPK-mediated negative regulation of mTORC1 could be completely blocked by AKT [30]. Adding to the complexity is that Redd1 seems to interact with hypoxia-induced factor α (HIF-α) under certain conditions [31,32]. Given that HIF-α, in turn, is activated by mTOR, the increased PI3K/AKT/mTOR pathway might regulate Redd1 via mTOR-mediated upregulation of HIF-α [33]. Another AKT-dependent mechanism of mTOR regulation is the release of mTORC1 inhibitor PRAS40 from the complex. Phosphorylation of PRAS40 at T246 by Akt and at S183/Ser221 by mTORC1 results in its dissociation and increasing of mTORC1 signaling [34,35]. The upstream effectors of mTOR are summarized in Table 1.

Unlike mTORC1, mTORC2 (as PDK2) is able to phosphorylate AKT at S473 to complete its full activation. Recent findings support the hypothesis about the positive feedback loop between AKT and mTORC2. AKT initiates mTORC2 activation by phosphorylating its subunit mSin1 at T86 site [36]. Therefore, the mechanism of AKT/mTORC2 activation appears to be the following: AKT is phosphorylated by PDK1 at T308, increased AKT phosphorylates mSin1 at T86, enhanced mTORC2 phosphorylates AKT at S473, Akt is fully activated. Although there are debates about the physiological importance of phosphorylation at T308 over the S473 site, these two events are certainly independent from each other. Interestingly, the activity of AKT-T308 phosphorylation alone may be sufficient for a subset of its physiological roles while AKT-S473 phosphorylation is required for other functions [37,38].

### 1.2. Structural and Functional Differences between mTORC1 and mTORC2

mTORC1 implements its regulatory role in protein synthesis through downstream targets, eukaryotic translation initiation factor 4E binding protein 1 (4E-BP1), and S6 kinase (S6K, also known as p70 ribosomal S6 kinase or p70S6K) [39]. It activates S6K resulting in upregulation of S6 and eukaryotic initiation factor-4B (eIF4B) but suppresses 4E-BP1, which leads to the dissociation of 4E-BP1 from eIF4E (Figure 1). Then eIF4E binds with eIF4G to promote translation. mTORC1 is also involved in cell proliferation by positively controlling the synthesis of lipids, essential components of the plasma membrane, via the sterol regulatory element binding protein 1/2 (SREBP1/2) and in autophagy by regulating its suppressor, death-associated protein 1 (DAP1) [40,41,42]. mTORC1 can stimulate a number of well-known PH-related pathways such as HIF-1α, Notch, nuclear factor kappa B (NF-κB), the signal transducer and activator of transcription 3 (STAT3), peroxisome proliferator-activated receptor γ (PPARγ), yin-yang 1-PPARγ coactivator-1α (YY1-PGC1α), the bHLH leucine zipper transcription factor EB (TFEB) [43,44,45,46,47,48,49]. Compared to mTORC1, much less is known about mTORC2 signaling network. mTORC2 directly activates serum- and glucocorticoid-induced protein kinase 1 (SGK1), a kinase controlling ion transport and growth [50]. Forkhead box O1/3a (FoxO1/3a), an inflammatory responsive transcription factor, has been established as another mTORC2 target [51,52]. Its positive regulation is realized via AKT-S473 phosphorylation. mTOR downstream targets are summarized in Table 1 and will be discussed below.

Besides the divergent upstream and downstream molecules, mTOR complexes interact with each other. The crosstalk between the complexes is not well investigated but the studies using gain- or loss-of-function approaches towards each of the complexes in different cell types are able to shed the light on mTORC1/mTORC2 regulation. Thus, Raptor silencing in mouse hepatocytes or rapamycin treatment in human mesenchymal stromal cells resulted in the increased phosphorylation of AKT-S473 and insulin-mediated activation of mTORC2 but not mTORC2 level [53,54]. However, Raptor knockdown did not affect mTORC2-specific AKT-S473 phosphorylation in PASMC isolated from IPAH patients [12]. The Rictor-mTOR complex is increased while the Raptor-mTOR complex is decreased in the setting of enhanced AMPK activity [53]. Vice versa, mTORC1 stimulation inactivates mTORC2 through the inhibition of mSin1 by S6K, which leads to mSin1 dissociation from mTORC2 [55].

The findings from mouse fibroblasts arguing against the idea about negative regulation between mTOR complexes because *Rictor* knockout (KO) did not affect TSC2, Raptor, and S6K [38]. In addition, Rictor overexpression in mesenchymal stromal cells had no effect on mTORC1 signaling determined by S6K level [54]. Nevertheless, in human fibroblasts and IPAH-PASMC, mTORC2 appears to be an upstream effector of mTORC1, since Rictor silencing diminishes the phosphorylation of S6K [12,56]. Available published literature confirms the complexity of the crosstalk between mTORCs indicating its dependency on cell type and/or experimental conditions.

The activity and function of mTOR is tightly coupled to its intracellular localization. When the level of amino acids is low, mTOR is mainly located in the cytoplasm. Upon growth factors stimulation, it is recruited to lysosomal membranes, however the pools of mTORC1 are also found in mitochondria and nucleus [57]. mTORC2 appears to exist at least in two subpopulations: Plasma membrane-localized pool promotes AKT activation in response to growth factors while lysosomal pool controls the efficiency of autophagy [58]. However, Betz et al. speculated that mitochondria-associated endoplasmic reticulum membranes appears to be mTORC2 signaling hub [59].

### 1.3. AKT/mTOR and PASMC Proliferation

Published observations support the importance of AKT/mTOR pathway in pulmonary vascular remodeling and the development of PAH. Concentric PA wall thickening due to excessive PASMC growth/proliferation and migration is one of the pathological hallmarks of the pulmonary vascular remodeling in patients and animals with PH. The pro-proliferative and pro-survival effect of AKT/mTOR in PASMC has been demonstrated in humans including patients with idiopathic PAH (IPAH) and chronic thromboembolic pulmonary hypertension (CTEPH), as well as in animal PH models, including hypoxia-induced PH (HPH), hypoxia/Sugen-induced PH (Su/Hyp-PH), and monocrotaline-induced PH (MCT-PH) [60,61,62,63,64,65,66,67]. The activated mTOR signaling determined by the increased protein levels of phosphorylated AKT (pAKT-S473 and pAKT-T308), phosphorylated mTOR (pmTOR), and its downstream targets has been reported in PASMC in these types or forms of PH. Accumulating reports provide a strong evidence indicating that both mTOR complexes play a pathogenic role in the enhanced PASMC proliferation and pulmonary vascular remodeling in PH.

Genetic approach in animal experiments has demonstrated the importance of the mTOR pathway in the development of PH (Table 2). Thus, mice with SMC-specific KO of *Pten* (*Pten*^SMC−/−^ mice) manifest spontaneous PH and RV hypertrophy as early as 20 days after the birth [68]. Compared with WT littermates, *Pten*^SMC−/−^ mice had the increased PA wall thickness, occluded precapillary arterioles, and enhanced autocrine SMC growth. Another group reported features of spontaneous PH in mice with inducible KO of *Pten* in SMC at the time of tamoxifen injection although RVSP was not significantly changed [69]. After exposure to hypoxia for four weeks, these mice displayed more severe PH compared with wildtype controls. The activated AKT signaling has been detected in PA, heart, and lung tissues from *Pten*^SMC−/−^ mice, but downstream effectors including mTOR were not studied. On the other hand, *Pten* overexpression resulted in the attenuated PH and vascular remodeling in transgenic mice exposed to hypoxia for four weeks [14]. The exact role of each AKT isoforms yet to be discovered, however we previously reported that mice with global KO of *Akt1*, but not *Akt2*, were protected against the development of HPH due to attenuated pulmonary vascular remodeling [14]. Moreover, hypoxia-induced proliferation of PASMC isolated from *Akt1*^−/−^ mice was inhibited in comparison with cells from WT or *Akt2*^−/−^ mice.

mTORC1 substrates S6K and 4E-BP are considered as the canonical molecular mechanisms for the increased proliferation of PASMC resulting in distal arteriole muscularization in HPH [48,66,82,83,84]. Right ventricular hypertrophy, predominantly due to increased afterload or increased pulmonary vascular resistance in Su/Hyp rat model of PH, has also been recently associated with upregulation of mTORC1 not mTORC2 signaling [85]. Multiple reports showed that many altered upstream pathways mediate signal transduction via mTORC1 to promote PH. Thus, mTORC1 activation in mice due to SMC-specific deletion of *Tsc1* (*Tsc1*^SMC−/−^ mice) is necessary and sufficient to increase PASMC proliferation and PA thickness leading to the development of spontaneous PH under normoxic conditions [75]. Marked activation of mTORC2, pAKT-S473, and glycogen synthase kinase 3 (GSK3) was detected in PA isolated from *Tsc1*^SMC−/−^ mice. Although there is a lack of direct evidence whether GSK3 is a shared target for Rictor and Raptor in PASMC, it appears to be regulated by both complexes in opposite directions in axon regeneration process: In contrast to pAKT-T308, mTORC2 and pAKT-S473 suppressed GSK3 phosphorylation [86]. Lee et al. also showed that downregulated TSC1 promoted mTORC1/S6K pathway under hypoxic conditions [87]. Global deficiency of another mTORC1 upstream molecule, *Ampkα2* (an AMPK isoform), resulted in the exacerbation of HPH due to excessive PASMC proliferation (Table 2) [72,88]. mTOR phosphorylation was higher in *Ampkα2*^−/−^ mice compared with WT mice under hypoxic conditions indicating the possibility for further activation of the mTOR pathway.

Some experiments were performed to identify the specific role of mTORC1 component, Raptor, in PA medial thickening. Thus, the results by Aghamohammadzadeh et al. showed that Raptor is required for aldosterone-induced survival pattern of PASMC defined by enhanced proliferation, viability, and apoptosis resistance through mTORC1/S6K pathway in Su/Hyp-PH and MCT-PH rats [89]. Our study on mice (at the age of 8–10 weeks) with SMC-specific deletion of *Raptor* or *Rictor* (*Raptor*^SMC−/−^ or *Rictor*^SMC−/−^) exposed to chronic hypoxia for three weeks also showed the predominant pathogenic role of mTORC1 in the concentric PA thickening and the development of HPH [15]. All these data strongly support the involvement of mTORC1 signaling in the regulation of PASMC proliferation and survival during PH.

Nevertheless, the role of mTORC2 should not be ruled out since both complexes are activated and contribute to PASMC proliferation in animals with HPH and MCT-PH and patients with IPAH [12,60,62,90]. Goncharov et al. demonstrated that mTORC2 promotes cell proliferation and survival via mTORC1 activation in AMPK-dependent manner [12]. Interestingly, neither Raptor nor Rictor siRNA affected normal PASMC proliferation. The transgenic mice with serotonin transporter (*5HTT*) overexpression in SMC exhibit the mTOR profile similar to hypoxic mice characterized by marked increases in mTORC1 and mTORC2 substrates [75]. Although other studies suggest that serotonin realizes its mitogenic effects on PASMC through AKT/mTORC1/S6K, it is still unclear whether mTORC1 activation precedes mTORC2 activation in *5HTT*-transgenic mice [91,92]. In contrast to this, disturbed mTORC2 pathway is able to stimulate cell proliferation under certain conditions. Additionally, older *Rictor*^SMC−/−^ mice (at the age of 3–6 months) develop spontaneous PH under normoxic conditions due to increased PDGF receptors and PA muscularization [15]. It is worth to note that pAKT-S473 was decreased while pAKT-T308 was not affected in PA isolated from older *Rictor*^SMC−/−^ mice suggesting the independence between these two events.

Pharmacological experiments in animal models of PH demonstrated the therapeutic potential to target AKT/mTOR signaling to prevent or inhibit the development of experimental PH, or reverse established PH (Table 2). A low dose of multikinase inhibitor, sorafenib (10 mg/kg per day orally administered for 14 days), was able to partially reverse RVSP and RV hypertrophy in rats with MCT-induced PH [76,77]. In addition, sorafenib improved vascular remodeling by reducing the number of fully muscularized PAs and restoring the proliferation/apoptosis ratio in PASMC. Interestingly, the beneficial effect of sorafenib on hemodynamics and morphological parameters was better than that of imatinib (RTK inhibitor). Nevertheless, the specific and selective effect of sorafenib or imatinib on AKT/mTOR expression and/or mTORC1/mTORC2 activity in vivo remains unknown.

AMPK activators (metformin and AICAR) were also therapeutically beneficial in animals with experimental PH [78,79,80]. Metformin improved survival in MCT-PH rats by 40%. Both drugs decreased PA cell proliferation and pulmonary vascular remodeling while metformin additionally had a therapeutic effect on endothelial function and pulmonary vasoconstriction since it increased carbachol-induced relaxation and reduced phenylephrine-induced contraction of PA rings isolated from hypoxic rats [78]. Dean et al. specifically tested metformin in female rats with Su/Hyp-PH and found it suppressed estrogen synthesis and metabolism [79]. These findings opened new avenues to investigate further therapeutic effect of metformin on animal models of PH in both females and males given that sex hormones including estrogen are considered as key mediators of sexually dimorphic features of PH in humans [93,94].

Rapamycin has been studied in vivo to treat PH for 20 years since the first report in MCT-PH rats [95]. At the dose of 1-5 mg/kg/day, it showed that rapamycin had a significant inhibitory effect on the development of HPH and MCT-PH in both prevention and reversal experiments due to attenuation of pulmonary vascular remodeling [60,66,81]. The authors observed that treatment with rapamycin was more potent than that of imatinib [60]. Moreover, one-week treatment with rapamycin, but not with imatinib, was also able to significantly reverse established PH in MCT rats. In contrast to imatinib, rapamycin normalized serum-stimulated proliferation of PASMC isolated from MCT-PH rats. Mechanistically, imatinib was also less efficient in reducing the levels of pAKT-S473, pAKT-T308, pGSK3, and pS6K in PASMC than rapamycin [60]. These data indicate that mTOR pathway could be therapeutically targeted by specific drugs to develop novel and effective treatments for patients with PAH and other forms of PH.

Moreover, AKT/mTOR has been established as a major mechanism for cell proliferation induced by growth factors (i.e., PDGF, FGF2) [67]. The activation of AKT/mTOR signaling was also linked to the enhanced PASMC proliferation induced by thrombin, blood-coagulation factor [96]. The phosphorylation of mTOR, AKT, and S6K is significantly increased in response to PDGF [65,97,98,99]. Besides, PDGF-BB increased the protein levels of either pAKT-S473 and pAKT-T308 in human PASMC supporting an idea that both mTOR complexes were activated by PDGF [90]. Additionally, IPAH-PASMC appear to be more sensitive to PDGF-BB since AKT-S473 and AKT-T308 phosphorylation in response to PDGF-BB are higher in IPAH-PASMC compared with normal PASMC. Nevertheless, the signal transduction associated with mTORC1 is considered as a key pathway for PDGF-induced PASMC proliferation. Thus, S6K inhibition or AMPK activation significantly reduced PDGF-induced proliferation [67,98,99]. Finally, short-term treatment with rapamycin markedly abolished PDGF effect on PASMC proliferation. It is worth to note that in our experiments, we failed to detect PDGF receptors in pulmonary arterial endothelial cells (PAEC) isolated from both normal subjects and IPAH patients probably due to very low level of expression [90]. Along with this, Ten Freyhaus et al. reported that PDGF-BB was not able to activate PDGF receptors in human coronary EC [100]. The findings led us to conclude that pro-proliferative effect of PDGF or thrombin via AKT/mTOR seems to be unique for PASMC since none of them increased AKT phosphorylation in human PAEC [96,99].

Given that Ca^2+^ is a major stimulus for cell contraction, proliferation, and migration, it is reasonable to propose the involvement of Ca^2+^ signaling in AKT/mTOR cascade. Indeed, PDGF induced a rise in cytosolic free Ca^2+^ concentration ([Ca^2+^]_cyt_) in PASMC [90]. In addition, PDGF also enhanced cyclopiazonic acid (CPA)-induced Ca^2+^ release from intracellular Ca^2+^ stores (SR/ER) and store-operated Ca^2+^ entry (SOCE) [65,99]. Published reports indicate that rapamycin is very effective in PH treatment due to its inhibitory effect on SOCE and suppression of PASMC proliferation (Table 2) [49,60,65,82,101]. Rapamycin also decreased PDGF-mediated upregulation of SOCE components, STIM1, and Orai1 [99]. In addition, blocking of Ca^2+^-sensing receptor (CaSR) with NPS2390 or transient receptor potential canonical 6 (TRPC6) with SKF96365 in human PASMC affected AKT/mTOR cascade but the authors did not specify the role of each complex in these experiments [102,103]. The upregulation of both CaSR and TRPC6 in PASMC contribute to the enhanced [Ca^2+^]_cyt_ and pulmonary vascular remodeling during PH in our published studies [16,104,105,106]. Besides, PDGF was unable to realize its stimulating effect on [Ca^2+^]_cyt_ in the presence of BAPTA-AM, a membrane-permeable Ca^2+^ chelator that can accumulate in the cytosol and intracellular organelles to chelate intracellular free Ca^2+^ [107,108]. These data shed a great light on the role of Ca^2+^ signaling in AKT/mTOR-mediated PASMC proliferation in PH.

mTOR-mediated proliferation is also associated with other critical signaling involved in the development of PH. For example, PDGF can upregulate HIF-1α protein expression, transcription factor controlling PASMC proliferation [97]. It indirectly supports the idea about the role of AKT/mTOR/HIF-1α axis in PDGF-mediated proliferation [64,109]. Indeed, mTORC1 can specifically activate HIF-1α signaling [45]. However, another study proposed HIF-1α as a downstream target of Rictor (or mTORC2) but not Raptor (or mTORC1) [12]. Notch is another major pro-proliferative pathway activated by mTOR [49]. Inability of Notch inhibitor, DAPT (a γ-secretase inhibitor), to abolish the activation of mTORC1 pathway in mice with HPH suggest that mTOR is an upstream regulator of Notch. Utilizing mouse lung tissues in these experiments remains an open question about the validation of crosstalk between mTOR and Notch pathways in PASMC especially given the fact that Notch regulates mTORC1 in cancer cells [110,111,112]. Finally, hypoxia-induced mTOR promoted the phosphorylation of nuclear factor kappa-light-chain-enhancer of activated B cells (NF-κB) in PASMC, which contributes to the inflammation due to vascular injury in PH [48]. It is in line with earlier study by Furgeson et al. demonstrating that PTEN depletion in culture of SMC increased PI3K/AKT/mTOR and NF-κB activity [113]. Moreover, we previously revealed the pathogenic role of NF-κB in PDGF-induced PASMC proliferation [114]. The complicated cross-talk between mTOR and other signaling during the pathogenesis of PH provides a fertile ground for further studies to identify the novel and therapeutically effective targets for treatment patients with different forms of PH.

### 1.4. AKT/mTOR and Phenotypical Transition of SMC

It is believed that PAH patients are divided into two groups based on the acute response to vasodilators during pulmonary vasodilator test: “responders” (or vascular responsive-PAH) and “non-responders” (vascular non-responsive-PAH) [115]. Pulmonary vasodilator testing is used to determine the relative contribution of reversible vasoconstriction versus vascular remodeling to choose the effective therapy. If the component of the reversible vasoconstrictive is significant, it identifies patients who may potentially benefit from long-term use of Ca^2+^ channels blockers to reduce PVR and PAP [116]. The positive dilation test is not observed very frequently in adults (about 10–15%) while pediatric patients respond in 40% cases [117,118,119,120]. Only half of “responders” in both groups benefit from long-term therapy with Ca^2+^ channels blockers [120]. The potential explanation for this phenomenon is the ultrastructural changes in remodeled vessels and severity of disease between “responders” and “non-responders”. We believe that at the early stage, or initiation of disease, the sustained pulmonary vasoconstriction is the major cause for the elevated PVR/PAP when a patient could be defined as a “responder” to pulmonary vasodilator test. Then, at the late stage, or progression of disease, the sustained vasoconstriction is replaced or dominated by vascular remodeling characterized by the adventitial, medial, and intimal hypertrophy of the pulmonary arteries, the intraluminal obliteration and occlusion of small pulmonary arteries and arterioles, and the neointimal and plexiform lesions in the pulmonary vasculature when patients have symptoms or require medical/surgical treatment. This hypothesis has support from animal models of PH where the “early” changes of the vessels are always the increased vasoconstriction and vascular tone (within a couple of weeks), then ultrastructural changes take place in EC (e.g., via EndMT) and SMC (e.g., via contractile-to-proliferative phenotypic transition and enhanced cell growth/proliferation) to initiate “remodeling” or concentric wall thickening and occlusive intimal lesions (which may also include inflammation-associated intimal, medial, and adventitial lesions).

Recent studies suggest the molecular differences in “responders” and “non-responders”. Hemnes et al. concluded that vascular smooth muscle contraction genes were more enriched in the “responders” suggesting that sustained vasoconstriction at early stage of PH or in less severe forms of PH could be a result from the layer of differentiated PASMC in contractile state [121]. Contractile PASMC are responsible for the regulation of vascular tone due to their contraction ability. However extremely plastic PASMC can de-differentiate from contractile (or quiescent) to proliferative (or synthetic) phenotype in response to many stimuli, i.e., hypoxia or PDGF [122,123,124]. The concept of phenotypic switch leading to the excessive PASMC proliferation and PA thickening is well accepted in the development and progression of PAH [124,125,126]. We previously showed that PASMC de-differentiation is mediated, at least in part, by a switch in Ca^2+^ signaling [105]. Thus, contractile PASMC have the increased voltage-dependent Ca^2+^ entry while PASMC in proliferative state exhibit the enhanced receptor-operated and store-operated Ca^2+^ entry.

**Figure 2:**
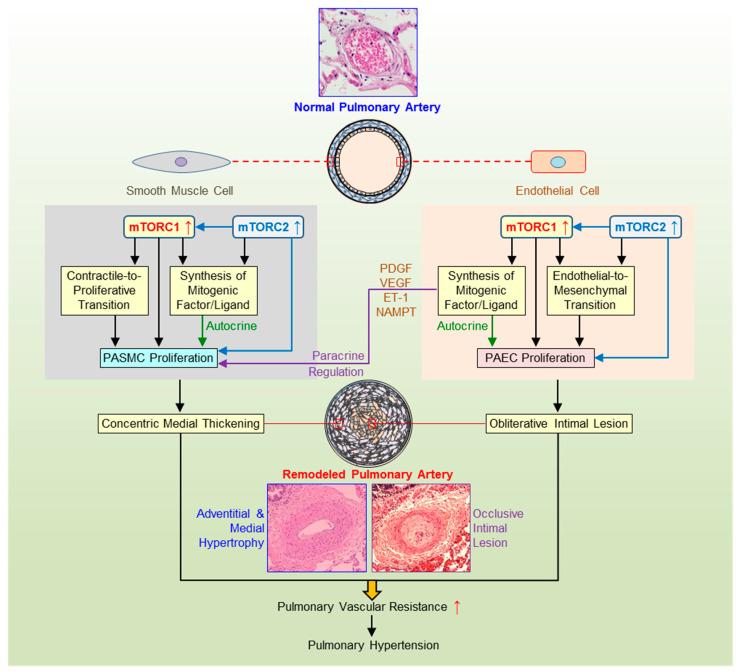
Pulmonary arterial smooth muscle and endothelial mTOR signaling in pulmonary vascular remodeling. Schematic diagram depicting the potential pathogenic role of the AKT/mTOR signaling in the development and progression of pulmonary hypertension (PH). Pulmonary vascular remodeling, such as concentric pulmonary arterial wall thickening due to pulmonary arterial smooth muscle (PASMC) proliferation and occlusive intimal lesions due to PASMC migration and pulmonary arterial endothelial cell (PAEC) injury and endothelial-to-mesenchymal transition (EndMT), is a major cause for the elevated pulmonary vascular resistance (PVR) and pulmonary arterial pressure (PAP) in patients with pulmonary arterial hypertension (PAH) and in animals with experimental PH. Activation of AKT/mTOR signaling or increased mTORC1 and mTORC2 activity in PASMC promotes contractile-to-proliferative phenotypic transition and enhances PASMC proliferation, which result in concentric pulmonary vascular wall medial hypertrophy and thickening. Activation of AKT/mTOR signaling or increased mTORC1 and mTORC2 activity in PAEC promotes EndMT, while EndMT-derived fibroblasts are involved in the development of occlusive intimal lesions and neointimal formation resulting in obliterative occlusion in small vessels. The mTORC1/mTORC2-mediated increases in gene expression and protein translation in PASMC and PAEC also enhance the synthesis and release of mitogenic factors (e.g., PDGF, VEGF, NAMPT) and ligands (e.g., ET-1) and stimulate PASMC/PAEC proliferation via autocrine and/or paracrine mechanisms. The mTORC1/mTORC2-mediated upregulation of membrane receptors, ion channels, and inflammatory ligands is also involved in the development concentric medial thickening and obliterative intimal lesions. PDGF, platelet-derived growth factor; VEGF, vascular endothelial growth factor; NAMPT, nicotinamide phosphoribosyltransferase; ET-1, endothelin-1.

The studies on SMC plasticity demonstrated that AKT/mTOR pathway appears to trigger cell de-differentiation [127,128,129]. The decreased expression of PTEN in SMC has been recently established as an initial event promoting phenotypic switch through the activation of serum response factor, transcription factor controlling SMC contractile gene expression [70]. Earlier, the same group generated *Pten*^SMC−/−^ mice, which manifest spontaneous PH due to severe SMC hyperplasia [68]. The nuclear HIF-1α expression was increased in SMC isolated from *Pten*^SMC−/−^ mice confirming AKT/mTOR-mediated regulation of HIF-1α. These findings are in line with the investigation by Houssaini et al. on *Tsc1*^SMC−/−^ mice, which developed marked pulmonary vascular remodeling characterized by higher density of muscularized pulmonary vessels and PA thickness due to loss of SMC contractile phenotype [75]. The authors linked the lack of contractile protein myosin heavy chain 11 with mTORC1/S6K activation. The published literature provided a compelling evidence that mTORC1/S6K pathway stimulated SMC de-differentiation while rapamycin restored contractile morphology and decreased collagen synthesis by 40–60% [127,130,131,132,133]. Furthermore, Zhan et al. proposed AMPK/TSC2/mTORC1/S6K molecular mechanism in osteoblastic differentiation model of vascular SMC induced by β-glycerophosphate [133]. Taken together, these data determined the critical role of mTORC1 complex in SMC phenotypic transition (Figure 2).

The knowledge about the involvement of mTORC2 in this process is limited; nevertheless, a few reports suggest the opposite roles of two complexes in SMC transition towards synthetic state. Martin et al. proposed the mechanism by which rapamycin promotes vascular SMC differentiation [131]. The phosphorylation of AKT-S473 was correlated with the restoration of contractile protein expression in SMC treated with rapamycin. Moreover, pre-treatment of cells with IGF-1 resulted in the enhancement of rapamycin effect indicating the potential activation of mTORC2. Similar observations came from the study by Hayashi et al. showing that the treatment of vascular SMC with IGF-1 led to the maintenance of differentiated phenotype by triggering PI3K/AKT cascade [134]. The recent publication based on in vivo and in vitro studies demonstrated that mTORC1 and mTORC2 differently orchestrate cell-fate program in mesenchymal stromal cells [54]. Rapamycin prevented osteoblast differentiation and calcification of mesenchymal stromal cells due to inhibition of mTORC1/S6K pathway and stimulation of mTORC2/AKT-S473 signaling. These results shed the light on SMC phenotype transition, which is mediated by the existing negative regulation between two mTOR complexes, but the exact mechanism has to be determined.

### 1.5. AKT/mTOR in SMC-EC Communication

The interaction between EC and SMC constantly exposed to the close contact and paracrine regulation are fundamental for the pathogenesis of diverse cardiovascular diseases including PH. The very early studies provided the first evidence that EC promotes SMC differentiation from synthetic to quiescent state by changing morphology, gene expression pattern, and cell contractility [135,136,137,138]. However, the specific paracrine mechanism responsible for the phenomenon was not identified at that time. Later, Brown et al. revealed the transition from proliferative to contractile phenotype in both the EC/SMC bilayer co-culture and SMC cultured alone in the conditioned medium collected from co-cultured EC/SMC [139]. The SMC differentiation was associated with the rapid phosphorylation of AKT-S473 (mTORC2 target) and the subsequent upregulation of differentiation protein markers. The conditioned medium collected from EC or SMC cultured alone did not induce SMC differentiation. In contrast, the conditioned medium from EC promoted SMC proliferation, migration, and protein synthesis [140]. In addition, it is a well-known fact that PDGF promotes SMC phenotypic transition from contractile to proliferative phenotype but some investigators speculated about the ability of PDGF to maintain SMC differentiation [134,141,142,143]. The analysis of published literature led us to conclude that it is not just one-way controlling SMC phenotype by AKT/mTOR pathway but rather a balancing differentiation/de-differentiation process depending on the specific conditions.

SMC-EC communication in PH has been also demonstrated in vivo, for example, in *Pten*^SMC−/−^ mice with enhanced susceptibility to severe HPH [69]. Animals exhibited occlusive vascular lesions characterized not only by medial thickening but also by plexiform intimal lesions due to hyperproliferative EC, a hallmark of human PAH but rarely observed in mouse HPH model. On the other hand, EC-specific deletion of *Pdk1* gene in mice was able to improve HPH via the inactivation of AKT/mTORC1 pathway in PA tissue and the attenuation of PA muscularization [71]. In our experiments, conditional and inducible KO of *Rictor* in EC failed to protect mice from the development of HPH suggesting a negligible role of endothelial mTORC2 (Table 2) [15]. Additionally, Omura et al. reported that normal mouse PASMC had the increased proliferation after stimulation with the conditioned medium collected from ex vivo incubation with lungs (predominantly consisted of EC [144]) isolated from mice with HPH [74]. Although the profile of endothelial AKT/mTOR remained mostly unknown, these studies provided the evidence about AKT/mTOR-mediated communication between EC and SMC in the development of PH (Figure 2).

### 1.6. AKT/mTOR and EC Dysfunction

Our knowledge on AKT/mTOR signaling mainly comes from studies on SMC function, while little is known about the role of endothelial mTOR in PH. Two recent independent studies on mice with constitutive deletion of *Ampk* or its isoform *Ampkα2* in EC (*Ampk*^EC−/−^ or *Ampkα2*^EC−/−^ mice) demonstrated that endothelial mTORC1 contributes to the pulmonary vascular remodeling and development of experimental PH (Table 2) [73,74]. The first in vitro reports appeared on the role of mTOR signaling in EC exposed to hypoxia. Thus, Li et al. performed time-course experiments using rat aortic EC treated with hypoxia for 24 h and concluded that hypoxia-induced EC proliferation required both mTOR complexes [145]. Interestingly, mTORC1 activation at early response to hypoxia precedes stimulation of mTORC2 axis. Consistently, overexpression of mTOR was sufficient to enhance proliferation of rat aortic EC under normoxia or hypoxia [146]. Moreover, the conditioned medium collected from hypoxic PAEC had higher concentrations of PDGF isoforms and mitogenic effect on PASMC measured by [^3^H]-thymidine incorporation technique and flow cytometric analysis [147]. The similar pro-proliferative effect was observed using the conditioned medium collected from human umbilical vein endothelial cells (HUVEC) after hypoxic exposure but it was not due to release of PDGF, indicating the potential role of other growth factors [148].

These data are in line with other studies as higher protein expression of PDGF-BB was detected in human or bovine PAEC exposed to hypoxia [149,150].

We also recently reported that the proliferation rate is higher in normal or IPAH PASMC cultured in the conditioned medium collected from PAEC due to PDGF released to the medium [90]. PDGF isoforms concentration detected by ELISA assay was significantly higher in the conditioned medium collected from IPAH PAEC compared with normal PAEC. Besides, the conditioned medium from PAEC was able to activate PDGF receptors and AKT/mTOR signaling in both normal and IPAH PASMC. These data are in line with the previous in vivo studies using HPH and MCT-PH models in rats demonstrating the upregulation of PDGF receptors and isoforms in lung tissue and higher concentration of PDGF-AA and PDGF-BB in plasma isolated from PH rats [151]. In addition, PDGF-BB level in lung tissues and PASMC proliferation were increased in *Ampk*^EC−/−^ mice with the exacerbated PH after exposure to hypoxia [74]. It is worth noting that the conditioned medium prepared from ex vivo incubation with lungs from hypoxic *Ampk*^EC−/−^ mice promoted normal mouse PASMC proliferation compared to hypoxic WT lungs. Overall, these findings imply that PAEC stimulate PASMC proliferation in a paracrine manner due to, at least, PDGF release and activation of SMC AKT/mTOR signaling, leading to medial thickening and pulmonary vascular remodeling in PH (Figure 2).

In addition to the promoting effect on PASMC proliferation and migration via a paracrine mechanism and direct cell-to-cell interaction through gap junctions and Notch signaling, EC also contribute to the development of PH by undergoing EndMT, the conversion from a fully differentiated, slowly growing phenotype to a highly proliferative, mesenchymal (myoFB or SMC-like) morphology [152,153]. EndMT has recently been established as an important component of PH phenotype [8,9,154]. At the molecular level, EndMT is characterized by activating key drivers such as Snai1 (Snail), Snai2 (Slug), Twist, ZEB, and switching from EC specific gene pattern to mesenchymal phenotype [153,155]. The elegant study by Song et al. explored whether PDGF signaling is involved in EndMT program using in vivo and in vitro approaches [151]. Mechanistically, the increased protein level of PDGF-A and PDGF-B in PAEC was correlated with EndMT induced by hypoxia. The treatment of PAEC with either PDGF-AA or PDGF-BB for 72 h resulted in EndMT promotion however PDGF-BB exhibited higher potency to induce EndMT. Furthermore, PDGF-BB siRNA attenuated hypoxia-mediated EndMT pattern. EndMT observed in different models of PH was inhibited by imatinib, PDGFR antagonist, due to upregulated CD-31 and downregulated fibronectin and vimentin [151,153,156,157].

Both PDGF-AA and PDGF-BB increased TGF-β_1_ expression in PAEC but TGF-β_1_, a well-known promoter of EndMT, enhanced PDGF-BB but not PDGF-AA in PAEC suggesting the positive reciprocal regulation between PDGF-BB and TGF-β_1_ [151]. Unfortunately, the authors did not attempt to investigate the role of AKT/mTOR in these experiments. Abundant evidence from the published literature demonstrated that components of AKT/mTOR signaling are critical factors for epithelial-to-mesenchymal transition (EMT) during carcinogenesis but some studies also shed a light on the relationships between AKT/mTOR and EndMT [158,159,160,161,162]. Thus, 48-h treatment of HUVEC with TGF-β_1_ led to the significant enhancement of EndMT, motility, and migration ability along with the activation of mTORC1/S6K axis [163]. In rats with HPH, the increased TGF-β_1_ inactivated PTEN to trigger PASMC proliferation and resistance to apoptosis via AKT signaling contributing to pulmonary vascular remodeling [164]. In lung FB, TGF-β launched the metabolic reprogramming by driving mTORC1 [165]. Hypoxia-mediated EndMT provoked mTORC1 phosphorylation in PAEC while the treatment with recombinant human BMP-7 markedly suppressed both events [166]. It is worth to note that BMP-7 deficiency was enough to induce spontaneous EndMT and PAEC migration under normoxic conditions but further investigations would be needed to define the cross-talk between mTOR and TGF-β family. In contrast, in mammary epithelial cells knockdown or pharmacological inhibition of mTORC1 triggered EMT characterized by the induction of the mesenchymal markers such as fibronectin, vimentin, together with the repression of epithelial markers such as E-cadherin [167]. The authors proposed the underlying mechanism as TGF-β-independent. In addition, two independent studies established the AKT/mTOR/S6K pathway as a necessary positive mediator for bleomycin-induced EndMT as bleomycin failed to transform HUVEC after treatment with rapamycin [168,169].

Although it is still unclear how mTORC1 and its downstream targets regulate EndMT, there are some speculations about the regulation of Snail and Slug by mTORC2. At first, Rictor silencing prevented human PAEC from undergoing phenotypical changes linked to EndMT induced by interleukin-13 [170]. Secondly, knockdown of Rictor but not Raptor enhanced Snail proteasomal degradation leading to its reduced expression in GSK3-dependent manner [171]. Thirdly, IGF-1 treatment significantly decreased expression of E-cadherin due to upregulated Snail and Slug but had no influence on Twist and ZEB1 levels in human ovarian cancer cells [172,173]. Rapamycin successfully reversed EMT by restoring E-cadherin [173,174]. Lastly, Rictor shRNA did not alter E-cadherin level while Raptor shRNA reduced E-cadherin expression [173]. Moreover, *RAPTOR*-deficient cells exhibit the increased pAKT-S473 and suppressed GSK3 along with the activation of E-cadherin transcriptional repressors such as Snail, ZEB1 and Twist resulted in EMT.

Furthermore, the new elegant study by Mammoto et al. provided strong evidence that EndMT in PAEC and PDGF-mediated excessive PASMC proliferation could be interdependent in PH [175]. The authors found that *TWIST1* overexpression increased PDGF-B protein in human PAEC. The most excited findings are that the conditioned medium collected from *TWIST1*-overexpressed PAEC stimulated PASMC DNA synthesis and migration in response to PDGF while *TWIST1*-silenced PAEC exhibit the opposite effect. Additionally, endothelial Twist1 also mediated hypoxia-induced PDGF-B expression and DNA synthesis in PASMC culturing in the conditioned medium collected from hypoxic PAEC.

In summary, these results together demonstrated that EC could contribute to neointimal formation, intimal thickening, and distal PA obliteration during PH by two AKT/mTOR-dependent mechanisms: Directly—via transformation into mesenchymal cells with pro-proliferative and apoptosis-resistant abilities, and indirectly—through stimulation of SMC proliferation and migration in a paracrine manner by releasing growth factors (Figure 2). The new avenues opened for investigations would uncover the molecular mechanisms of the interaction between these two events, which is critical to our elucidation of the etiology and pathogenesis of PH.

### 1.7. AKT/mTOR Signaling as a Therapeutic Target for PH

The evidence from published research works has generated significant interest for targeting the components of AKT/mTOR pathway for PH therapy. The potential therapeutical agents for PH treatment could be from the group of RTK inhibitors (i.e., imatinib), PI3K inhibitors, AMPK activators, AKT inhibitors, and mTOR inhibitors (e.g., rapamycin), however not all of them are under development in clinical trials to treat PH.

Imatinib (sold under the brand name Gleevec^®^) was the first RTK blocker approved by FDA to treat cancer in 2001. As the first significant compound with the ability to reverse pulmonary vascular remodeling in animal experiments, it attracted great interest from PH community specialists [176]. Later, in 2005, the group of investigators reported a therapeutic potential of imatinib as an add-on in a 61-year-old patient with PAH who did not responded to the triple therapy (dual endothelin A and B receptor antagonist, bosentan; a phosphodiesterase 5 inhibitor, sildenafil; and an analog of prostacyclin, iloprost) [177]. It led to significant improvement in PVR (32%) and the 6-min walking distance (6MWD) test (47%). The results from the first randomized, double-blind, placebo-controlled trial became available in 2010 [178]. This Phase II study was designed to assess the safety, tolerability, and efficacy of imatinib in 59 patients with PAH (NCT00477269). Analysis of 42 patients that completed six-month therapy showed significant decrease in PVR and increased in cardiac output (CO); nevertheless, 6MWD was not changed in the imatinib group compared to the placebo. Unfortunately, adverse effects such as nausea, headache, and edema were observed in 11 patients in both groups (Table 3).

The similar results were received in two following-up clinical trials: The observation study in Switzerland on 15 patients and Phase III multinational study IMPRES on 202 patients (NCT00902174) [179,182,183]. Severe side effects including subdural hematomas in 4–5% cases led the sponsors to adjust the international normalized ratio from 2.5 to around 2.0, which limited the use of imatinib as add-on therapy in PAH [184].

Given the existence of cancer paradigm of PAH, it was expected that several clinical trials were initiated to test other RTK inhibitors approved as cancer therapeutics in the setting of PAH. Thus, nilotinib (PDGF receptor, c-Kit, and Abl antagonist) showed promising results in preclinical experiments on fos-related antigen 2 transgenic mice as novel model for systemic sclerosis-associated PAH [185]. Histological abnormalities such as intimal thickening with concentric laminar lesions, medial hypertrophy, perivascular inflammatory infiltrates, and adventitial fibrosis mimicked the main characteristics in humans and indicated the severity of the disease. Oral treatment with nilotinib for eight weeks resulted in strong improvement of proliferative vasculopathy and lung fibrosis. Unfortunately, a phase II clinical trial initiated in 2010 was prematurely terminated due to serious adverse effects including cardiac, gastrointestinal, and hepatobiliary disorders (NCT01179737).

Another multi-kinase inhibitor, sorafenib, which previously showed effectiveness in animal models of PH, was tested in clinical trials (Table 3) [76,77]. The first report on safety, tolerability, and exploratory efficacy evaluation of sorafenib in patients with PAH in 2010 demonstrated that sorafenib was well tolerated at 200 mg twice daily in 12 patients (NCT00452218) [180]. Although sorafenib generated a modest improvement, the authors did not recommend “compassionate” use of this drug for PAH treatment (Table 3). In fact, 30–40% patients displayed sorafenib-related manageable adverse events (hand-foot syndrome, rash, and alopecia). All participants were receiving parenteral prostanoid therapy, which made it difficult to extrapolate the safety for the determined dose regimen to patients with other PAH therapies or without any treatments. Recently, the small study by Kimura et al. confirmed favorable effects of sorafenib (100–400 mg/day) to improve symptoms in refractory PAH with similar tolerated adverse events [186].

Clinical experience with other RTK inhibitors such as nilotinib, bosutinib, lapatinib, ponatinib, carfilzomib, dasatinib, and ruxolitinib urges caution because they appeared to be associated with the development of subclinical PAH in the treatment of cancer patients [187,188,189,190]. Of these, dasatinib have the strongest documented ability to induce PAH [191,192]. Using echocardiography to estimate non-invasively the mean tricuspid regurgitation peak gradient, which reflects PAP, Minami et al. observed possible PAH onset in 10% and 13.2% patients with chronic myeloid leukemia (CML) treated with nilotinib and dasatinib, respectively [193]. Of note, 2.7% CML patients treated with imatinib also displayed drug-induced PAH. A new pharmacovigilance-pharmacodynamic study aimed to answer why RTK blockers paradoxically have been linked to both cause and treatment for PAH [190]. A positive disproportionality signal was found for dasatinib, bosutinib, ponatinib, ruxolitinib, and nilotinib indicating that the benefits unlikely outweigh the risks for use in humans. The authors highlighted potential roles of BMP signaling pathway and the Src protein kinase family in the pathophysiology of RTK-induced PAH. Undoubtedly, further exploring of the underlying mechanisms would provide important information to combat this rare and severe adverse event.

Promising results from animal studies with AMPK activator, metformin, provided the rationale to test its therapeutic effect in PAH patients. In a recent Phase II clinical trial, Brittain et al. demonstrated compelling evidence that metformin therapy was safe and well tolerated in 20 patients with PAH [181]. Although no changes were reported in RVSP and 6MWD, RV function and lipid deposition were significantly improved. Only one patient did not reach the target dose of metformin due to side effects. The most common side effects included diarrhea, heartburn, and abdominal pain while no serious adverse effects were documented. Another Phase II, randomized, blinded clinical trial on 130 subjects was initiated in 2018 to assess metformin effect on 6MWD, WHO functional class, RV function, and lipid content (NCT03617458).

Inhibition of mTOR by rapamycin (sirolimus, rapamune) appears to be a prime target for developing another strategy for PAH treatment. Rapamycin was purified from a soil bacterium Streptomyces hygroscopicus in the early 1970s and originally was proposed to be used as antifungal agent. Later, potent immunosuppressive and cytostatic anti-cancer activity was revealed, which received a significant interest from pharmaceutical companies to launch multiple clinical trials [194,195,196]. As an immunosuppressant, it was approved by FDA in 2003 for kidney transplantation. In addition, in 2010, Wessler et al. reported a case study on PAH improvement in a 61-year-old patient who received rapamycin treatment due to pancreatic cancer [197]. The results from the first pilot trial to evaluate the safety and efficacy of rapamycin analog, everolimus, to treat severe PAH became available in 2013 [198]. Out of 10 PAH patients (8 with IPAH and 2 with CTEPH) enrolled in the study, 8 completed everolimus therapy for 6 months. Besides two patients with acute bronchitis and the worsening of right heart function, no other adverse side effects were observed. In 87.5% of cases, PVR was decreased with the mean of 31%. Moreover, mean PAP and CO were also significantly improved while other parameters including 6MWD displayed only a trend, probably due to a small population. In 2017, a clinical trial was initiated to test ABI-009, albumin-bound rapamycin, on 25 PAH patients (NCT02587325). The first results expected in near future would help in developing the improved therapeutic options for the PAH patients, especially for those who do not respond to current therapies.

## 2. Summary and Conclusions

Despite expanding research into the diagnosis and treatment of PAH, death rates from this disease have continued to increase 2.5% per year for women and 0.9% per year for men during the past decade. Published data demonstrate that mTOR is essential for pulmonary vascular remodeling and occlusive intimal lesions, which suggests mTORC1/2 complexes, Raptor and Rictor, may both contribute to the development and progression of PAH. Targeting the pathway using specific inhibitors for PI3K, AKT, mTOR, Raptor, or Rictor is considered as a good therapeutic strategy to develop novel and specific treatment for PAH. Unfortunately, there are currently no available pharmacological compounds that are able to specifically inhibit mTORC1 or mTORC2. Based on our results, genetic ablation of both Raptor and Rictor using SMC-specific deletion of *mTOR* had the greatest inhibitory effect on PH development compared with *Raptor* (mTORC1) or *Rictor* (mTORC2) deletion alone [15].

Recently, small-molecule ATP-competitive mTOR inhibitors that block both mTORC1 and mTORC2 were developed [199,200]. Due to similar ATP sites in mTOR and PI3K, multiple PI3K inhibitors could act as dual inhibitors suppressing the activity of mTOR as well. It is of a great interest to test a powerful two-hit inhibition system in PH animal models given the fact that many dual PI3K/mTOR inhibitors emerged into clinical trials as anti-cancer agents [201]. More studies need to be done to answer three fundamental questions on the therapeutic design: (a) Whether the drug should target the more upstream proteins of the signaling pathway (e.g., PI3K, AKT) or the downstream proteins (e.g., mTOR); (b) whether the specific drug for mTORC1 (e.g., by inhibiting Raptor) or mTORC2 (e.g., by inhibiting Rictor) is more beneficial than blocking the upstream proteins or the “whole” signaling pathway (e.g., RTK, or PI3K), and (c) whether targeting both upstream and downstream regulator (e.g., RTK blocker and Raptor blocker) is therapeutically reasonable for PAH treatment.

Novel therapeutic targets are desperately needed for developing new drugs for treatment of PAH. Elucidating precise pathogenic mechanisms at levels of gene, molecule, cell, organ, and system will be definitely translated into novel therapeutic approaches for different forms of PAH.

## Figures and Tables

**Table 1:** The list of upstream and downstream targets of mTOR.

Target	Position	Regulation by mTOR	Role	Function
RTK	Upstream	---	Activates PI3K	mTOR↑
PI3K	Upstream	---	Converts PIP2 to PIP3	mTOR↑
PTEN	Upstream	---	Dephosphorylates PIP3 to PIP2	mTOR↓
PDK1	Upstream	---	Phosphorylates AKT at T308	mTOR↑
AKT	Upstream	---	Inhibits TSC1/TSC2	mTOR↑
TSC	Upstream	---	Inhibits RHEB	mTOR↓
RHEB	Upstream	---	Phosphorylates mTOR	mTOR↑
Redd1	Upstream	---	Activates TSC	mTOR↓
AMPK	Upstream	---	Activates TSC	mTOR↓
P70S6K	Downstream	Activation	Activates S6	PASMC growth and proliferation↑
			Activates eIF4B	Gene translation↑ Protein synthesis↑ PASMC growth and proliferation↑
			Inhibits MHC11	SMC contractile-to-proliferative transition↑
4E-BP1	Downstream	Inhibition	Inhibits eIF4E	Gene translation↑ Protein synthesis↑
SREBP	Downstream	Activation	---	Lipid synthesis↑
DAP1	Downstream	Activation	---	Autophagy↓
SGK1	Downstream	Activation	---	Ion transport↑ Cell apoptosis↑
FoxO1	Downstream	Inhibition	---	Inflammatory response↓
GSK3	Downstream	Inhibition	---	ER stress↓ Glycogen synthesis↓
			Inhibits Snail	EndMT↑
Slug	Downstream	Activation	---	EndMT↑
HIF-α	Downstream	Activation	---	Hypoxia response↑
NF-κB	Downstream	Activation	---	Inflammatory response↑
Notch	Downstream	Activation	---	Cell-cell interaction↑
PPAR	Downstream	Activation	---	Glucose metabolism↓ Lipid metabolism↑
STAT3	Downstream	Activation	---	Immune response↓
TFEB	Downstream	Activation	---	Lysosomal biogenesis↑ Autophagy↑
YY1	Downstream	Activation	---	Cell proliferation↑

**Table 2:** The list of in vivo studies demonstrating the role of AKT/mTOR signaling in the development of PH.

Target	Method	PH model	Effect	Parameters	Ref
**Genetic Approach**					
PTEN	Transgenic global overexpression	HPH in mice	Inhibition	Pulmonary vascular remodeling↓ RVSP↓ RV hypertrophy↓ PA wall thickness↓	[14]
PTEN	Conditional KO in SMC	Spontaneous PH in mice (at the age of 20 days)	Promotion	Pulmonary vascular remodeling↑ RV hypertrophy↑ PA wall thickness↑ SMC proliferation↑	[68]
PTEN	Conditional and inducible KO in SMC	---	---	Aorta contractility↓ SMC de-differentiation↑	[70]
PTEN	Conditional and inducible KO in SMC	Spontaneous PH in mice	Partial promotion	RVSP RV hypertrophy↑ Pulmonary vascular remodeling↑ PA wall thickness↑	[69]
		HPH in mice	Promotion	RVSP↑ RV hypertrophy↑ Pulmonary vascular remodeling↑ PA wall thickness↑	
PDK1	Conditional KO in EC (heterozygous)	HPH in mice	Inhibition	Pulmonary vascular remodeling↓ RVSP↓ RV hypertrophy↓ PA wall thickness↓	[71]
AKT1	Global KO	HPH in mice	Inhibition	RVSP↓ RV hypertrophy↓ Pulmonary vascular remodeling↓ PA wall thickness↓	[14]
AKT2	Global KO	HPH in mice	No effect	RVSP RV hypertrophy Pulmonary vascular remodelingPA wall thickness	[14]
AMPKα2	Global KO	HPH in mice	Promotion	RVSP↑ RV hypertrophy↑ Pulmonary vascular remodeling↑ PASMC proliferation↑	[72]
AMPKα2	Conditional KO in EC	Su/Hyp-PH in mice	Promotion	RVSP↑ RV hypertrophy↑ PA wall thickness↓ Pulmonary vascular remodeling↑	[73]
AMPK	Conditional KO in EC	HPH in mice	Promotion	RVSP↑ RV hypertrophy↑ PA wall thickness↓	[74]
TSC1	Conditional KO in SMC	Spontaneous PH in mice (at the age of 10–12 weeks)	Promotion	RVSP↑ RV hypertrophy↑ Pulmonary vascular remodeling↑ PA wall thickness↑ PASMC proliferation↑ SMC de-differentiation↑	[75]
mTOR	Conditional and inducible KO in SMC	HPH in mice	Inhibition	RVSP↓ RV hypertrophy↓ Pulmonary vascular remodeling↓ PA wall thickness↓ Polycythemia↓	[14,15]
Raptor	Conditional and inducible KO in SMC	HPH in mice	Partial inhibition	RV hypertrophy↓	[15]
Rictor	Conditional and inducible KO in SMC	HPH in mice (at the age of 8–10 weeks)	Negligible inhibition	RVSP RV hypertrophy	[15]
	Conditional and inducible KO in SMC	Spontaneous PH in mice (at the age of 6–8 months)	Promotion	RVSP↑ RV hypertrophy↑ PA wall thickness↑	
	Conditional and inducible KO in EC	HPH in mice (at the age of 8–10 weeks)	No effect	---	
**Pharmaceutical approach**					
Sorafenib (multikinase inhibitor)	Oral gavage (10 mg/kg/day)	MCT-PH in rats	Inhibition	RVSP↓ RV hypertrophy↓ Pulmonary vascular remodeling↓ PA wall thickness↓ PASMC proliferation↓ PASMC apoptosis↑	[76]
Sorafenib (multikinase inhibitor)	Oral gavage (10, 30, or 100 mg/kg/day)	MCT-PH in rats	Inhibition	RVSP↓ RV hypertrophy↓ Cardiopulmonary remodeling↓ Pulmonary vascular remodeling↓	[77]
Imatinib (RTK inhibitor)	Oral gavage (50 mg/kg/day)	MCT-PH in rats	Inhibition	RVSP↓ RV hypertrophy↓ Pulmonary vascular remodeling↓ PA wall thickness↓	[76]
Imatinib (RTK inhibitor)	Oral gavage (100 mg/kg/day)	MCT-PH in rats	Inhibition	PAP↓ RV hypertrophy↓ Pulmonary vascular remodeling↓ PA wall thickness↓	[60]
Metformin (AMPK activator)	Intraperitoneally (100 mg/kg/day)	HPH in rats	Inhibition	Mean PAP↓ RV wall thickness↓ RV hypertrophy↓ PFAT↑ PA contraction↓ PA cell proliferation↓ Endothelial function↑	[78]
		MCT-PH in rats	Inhibition	Mean PAP↓ RV hypertrophy↓ Survival↑	
Metformin (AMPK activator)	Oral gavage (100 mg/kg/day)	Su/Hyp-PH in rats	Inhibition	RVSP↓ RV hypertrophy↓ Pulmonary vascular remodeling↓	[79]
AICAR (AMPK activator)	Intraperitoneally (1 mg/kg/day)	HPH in rats	Inhibition	Mean PAP↓ RV hypertrophy↓ Pulmonary vascular remodeling↓ PA wall thickness↓	[80]
Rapamycin (mTOR inhibitor)	Oral gavage (5 mg/kg/day)	MCT-PH in rats	Inhibition	PAP↓ RV hypertrophy↓ Pulmonary vascular remodeling↓ PASMC proliferation↓	[60]
Rapamycin (mTOR inhibitor)	Oral gavage (1 mg/kg/day)	HPH in rats	Inhibition	Pulmonary vascular remodeling↓ RVSP↓mPAP↓ PA wall thickness↓	[66]
Rapamycin (mTOR inhibitor)	Intraperitoneally (3 mg/kg/day)	HPH in mice	Inhibition	RV hypertrophy↓ Pulmonary vascular remodeling↓ PA wall thickness↓	[81]

**Table 3:** The list of pharmaceutical drugs used in clinical trials for PAH patients.

Drug	Dose	N of Participants	Status	Results	Effect	Adverse Effects	Identifier
Imatinib RTK inhibitor)	---	130	Completed	Not available	---	---	NCT01092897
Imatinib (RTK inhibitor)	200–400 mg orally once daily	59	Completed (24-week randomized, double-blind, placebo-controlled)	Available [178]	PVR↓ CO↑	Nausea, headache, peripheral edema	NCT00477269
Imatinib (RTK inhibitor)	200–400 mg orally once daily	202	Completed (24-week randomized, double-blind, placebo-controlled)	Available [179]	PVR↓ mPAP↓ CO↑ 6MWD↑	Cardiac failure, subdural hematoma, dyspnea, worsening PAH	NCT00902174
Nilotinib (RTK inhibitor)	50–300 mg orally twice a day	23	Terminated (due to serious adverse events)	Not Available	---	Cardiogenic shock, right ventricular dysfunction, gastric ulcer hemorrhage, cholecystitis, hepatitis	NCT01179737
Sorafenib (multikinase inhibitor)	200–400 mg twice daily	12	Completed (16-week, open-label)	Available [180]	CO↓	Diarrhea, hand–foot syndrome, rash, alopecia	NCT00452218
Metformin (AMPK activator)	500 mg–1000 mg orally once or twice daily	1899	Recruiting	Preliminary [181]	RVSP 6MWD RV function↑ RV lipid content↓	---	NCT01884051
Metformin (AMPK activator)	1000 mg orally twice daily	130	Recruiting	Not available	---	---	NCT03617458
Rapamycin (mTOR inhibitor)	---	25	Recruiting	Not Available	---	---	NCT02587325
